# Radiological semantics discriminate clinically significant grade prostate cancer

**DOI:** 10.1186/s40644-019-0272-y

**Published:** 2019-12-03

**Authors:** Qian Li, Hong Lu, Jung Choi, Kenneth Gage, Sebastian Feuerlein, Julio M. Pow-Sang, Robert Gillies, Yoganand Balagurunathan

**Affiliations:** 1 Department of Radiology, National Clinical Research Center of Cancer, Key Laboratory of Cancer Prevention and Therapy, Tianjin Medical University Cancer Institute and Hospital, Tianjin, China; 2 Department of Cancer Physiology, H.Lee.Moffitt Cancer Center, Tampa, FL USA; 3 Department of Radiology, H.Lee.Moffitt Cancer Center, Tampa, FL USA; 4 Department of GenitoUrology, H.Lee.Moffitt Cancer Center, Tampa, FL USA; 5 Quantitative Sciences, Department of Biostatistics and Bioinformatics, H.Lee.Moffitt Cancer, Tampa, FL 33612 USA

**Keywords:** Prostate cancer, Pirads, Semantics, Radiological traits

## Abstract

**Background:**

Identification of imaging traits to discriminate clinically significant prostate cancer is challenging due to the multi focal nature of the disease. The difficulty in obtaining a consensus by the Prostate Imaging and Data Systems (PI-RADS) scores coupled with disagreements in interpreting multi-parametric Magnetic Resonance Imaging (mpMRI) has resulted in increased variability in reporting findings and evaluating the utility of this imaging modality in detecting clinically significant prostate cancer. This study assess the ability of radiological traits (semantics) observed on multi-parametric Magnetic Resonance images (mpMRI) to discriminate clinically significant prostate cancer.

**Methods:**

We obtained multi-parametric MRI studies from 103 prostate cancer patients with 167 targeted biopsies from a single institution. The study was approved by our Institutional Review Board (IRB) for retrospective analysis. The biopsy location had been identified and marked by a clinical radiologist for targeted biopsy based on initial study interpretation. Using the target locations, two study radiologists independently re-evaluated the scans and scored 16 semantic traits on a point scale (up to 5 levels) based on mpMRI images. The semantic traits describe size, shape, and border characteristics of the prostate lesion, as well as presence of disease around lymph nodes (lymphadenopathy). We built a linear classifier model on these semantic traits and related to pathological outcome to identify clinically significant tumors (Gleason Score ≥ 7). The discriminatory ability of the predictors was tested using cross validation method randomly repeated and ensemble values were reported. We then compared the performance of semantic predictors with the PI-RADS predictors.

**Results:**

We found several semantic features individually discriminated high grade Gleason score (ADC-intensity, Homogeneity, early-enhancement, T2-intensity and extraprostatic extention), these univariate predictors had an average area under the receiver operator characteristics (AUROC) ranging from 0.54 to 0.68. Multivariable semantic predictors with three features (ADC-intensity; T2-intensity, enhancement homogenicity) had an average AUROC of 0.7 [0.43, 0.94]. The PI-RADS based predictor had average AUROC of 0.6 [0.47, 0.75].

**Conclusion:**

We find semantics traits are related to pathological findings with relatively higher reproducibility between radiologists. Multivariable predictors formed on these traits shows higher discriminatory ability compared to PI-RADS scores.

## Background

Prostate cancer is the most prevalent cancer and the second cause of cancer deaths among men in the USA [1]. A reliable prostate cancer screening approach that provides accurate risk assessment for targeting, diagnosis and treatment is still a critical need. The European Randomized Study of Screening for Prostate Cancer (ERSPC) reported that PSA-based screening has reduced the rate of death from prostate cancer by 20% [2], but is limited by a low specificity leading to over diagnosis at an estimated rate of 23 to 42% [3].

Transrectal ultrasound (TRUS) -guided needle biopsy of the prostate is recommended for patients with elevated serum PSA levels, an abnormal feeling prostate on digital rectal examination, or both. Given the heterogeneous and multifocal nature of prostate cancer, both indolent and clinically significant tumors may be found in the same gland. It is also known that tumors located in certain regions of the prostate are under sampled, missing dominant or high-grade tumors in these regions. In addition, prostate cancer stage upgrading or downgrading frequently occurs following repeat biopsies [4]. More recently, ultrasound-MRI fusion guided needle biopsies have been shown to improve precision in identifying, targeting and sampling prostate lesions of interest [5, 6].

Multi-parametric magnetic resonance imaging (mpMRI) has shown great promise as a non-invasive approach for prostate cancer detection [7], but the lack of uniform interpretation and reporting has led to high variability among radiologists [8].

But it has been generally agreed that, radiological appearance and the following interpretable descriptions are related to cancer progression [9, 10]. Radiologist training in the performance, interpretation and reporting of prostate imaging studies plays a major role in improving the performance of cancer detection in prostate cancer [11]. Various groups have developed radiological-based reporting scales for prostate cancer [12–14]. For example, a *Likert* reporting scale has been recommended by the Prostate Diagnostic Imaging Consensus Meeting (PREDICT) panel, and quantifies radiologist(s) opinion to a simplified 5 point scale [15].

The European Society of Urogenital Radiology (ESUR) first proposed the use of the Prostate Imaging Reporting and Data System (PI-RADS) as a way to standardize reporting of imaging consensus criteria. PI-RADS was later adopted by the American College of Radiology (ACR), and jointly proposed changes were formulated in a revision of the criteria [16, 17]. Findings on mpMRI are assessed on a 5-point categorical scale, based on the expert’s observational probability that a combination of findings on T_2_-weighted (T_2_WI) sequences, diffusion-weighted MRI (DWI) and dynamic contrast-enhanced MRI (DCE-MRI) correlate with the presence of a clinically significant prostate cancer at the specific location. The overall PI-RADS score considers a combination of multiple features obtained for the modality/sequences, such as nodule shape, margin and intensity. The PI-RADS assessment categories have a range of 1 to 5, with 5 being most likely to represent clinically significant prostate cancer. Previous studies [18, 19] have shown moderate inter-reader agreement with PI-RADS. The major pitfall in the clinical use of PI-RADS has been the degree of subjectivity of radiologists in study interpretations, leading to large variability in reported findings, and a suboptimal ability to characterize the nature and/or degree of malignancy in a lesion of interest [20].

Locating and discriminating clinically significant from insignificant cancers remains a challenge in prostate cancer screening. Current validation is primarily based on the pathologic Gleason score. Patients with Gleason score ≥ 7 ((3 + 4) or (4 + 3)) are considered clinically significant forms of cancer with increasing aggressiveness as the score increases to 8, 9 and 10 [20]. Recently, there have been numerous efforts to develop quantitative metrics for medical imaging to identify and describe abnormalities in radiological studies [21–24].

In this study, we propose to describe radiological traits independently for each mpMRI sequence on a numerical point scale. These traits were then taken in combination and related to pathological outcome of cancer aggressiveness (Gleason score) using a linear classifier approach. These combinations of traits were rigorously evaluated in a cross validation setting with multiple repeats. The semantic-based feature model was then compared to PI-RADS based predictors at different cutoffs to find clinically significant grade cancer.

## Methods

### Patients cohort

The study was approved by the Institutional Review Board (IRB) at the University of South Florida, and patient informed consent was waived for the retrospective analysis. All the patients were referred to the Radiology department for multiparametric MRI and targeted prostate lesion biopsy planning using the UroNav Ultrasound-MR Fusion Biopsy System (Invivo Corporation) at the H. Lee Moffitt Cancer Center. The patients were scanned using a Siemens 1.5 T MRI scanner with endorectal coil placement (Table 1). The inclusion criteria were as follows: a) availability of at least one targeted biopsy by the UroNav fusion system identified on the original interpretation, b) availability of mpMRI sequences (T_2_WI, DCE, DWI/ ADC) suitable for PI-RADS (version-2) scoring, and c) no image related limitations; i.e., post-biopsy hemorrhage, motion artifacts, et al.

**Table 1:** Clinical characteristic of the study cohort

a. Biopsy Histology characterization			
	Category	Total Number Biopsies: 167 (103 Patients)	Over-read Cohort (random): (40 Biopsy, 34 Patients)
1	Benign	77 (53 patients)	19
2	Gleason (3 + 3): 6	33 (28 patients)	8
3	Gleason (3 + 4 or 4 + 3):7	45 (35 patients)	11
4	Gleason: 8 or 9	12 (9 patients)	2
b. Patient characteristics of the study cohorts.			
	Category	Total Number (103 Patients)	Over-read Cohort: (random) (32Patients)
1	Age (Median, range)	67 [44, 83]	65.53, [52,76]
2	Race	White:98, Black:2, Others:3	White: 30, Others: 2.
3	Disease Grade	T1a:23,T1b:2,T1C:59,T2A:3, T2C:1,T3A: 3, T3B:1: Benign:15	T1:1, T1C: 21, T2A: 2, T3a: 1, Benign/Others: 7
4	Gleason Grade	GS≥6: 90, (=6,33; =7,45; =8,4; =9,8) Benign: 77	GS ≥ 6: 19 (=6:6,=7,10,=8,2,=9,1) Benign: 13 .
5	PSA (ng/ml)	7.56, [0.64, 44.7]	6.63 (6.59), [2.39,15.95]
6	Tumor Target Zone (Peripheral /Transition)	Central: 56 Peripheral:102 Middle: 9	Central: 16 Peripheral: 23 Middle: 1

The exclusion criteria include patients with prior localized treatment such as external beam radiation therapy, brachytherapy or cryoablation. The data curation step resulted in excluding 24 patients from the initial list, leaving 103 patients (167 biopsies) qualifying for the study cohort. We had 90 biopsies (65 unique patients) with Gleason scores ≥6, 33 of those biopsies had Gleason scores equal to 6, and 57 biopsies had Gleason scores ≥7. The rest of the 77 biopsies were negative for cancer (benign). Data extracted included age, race, smoking status, other cancer history, family cancer history, PSA level and board certified pathologist evaluated the cancer status and gleason scores for the slides. Multi-parametric MRI scans (T_2_WI, DCE, DWI, ADC) were downloaded from the Picture archive communication systems (PACS). Semantics were scored using offline DICOM (digital imaging and communication in medicine) viewers with prostate specific window settings.

### Radiologist marked biopsy targets

The clinical radiologist marked most aggressive target locations on the mpMRI scans and converged based on consensus reading with a fellow radiologist on duty. The markings were carried out using the commercial prostate biopsy system (Uronav/DynaCAD, Invivo inc, FL) that integrates the software modules to the biopsy hardware, that includes real time ultrasound (TRUS) location system. Patient preparation and endo-rectal coil placement follows the standard procedure. Using the automatic spring loaded biopsy-needles targeted core biopsies was obtained. Additionally, standard extended-pattern 12-core biopsies (Sextants) were obtained in accordance with the NCCN guidelines. The core targets were separately labeled and processed.

### Semantics and PI-RADS-version2 scoring

**Fig. 1:**
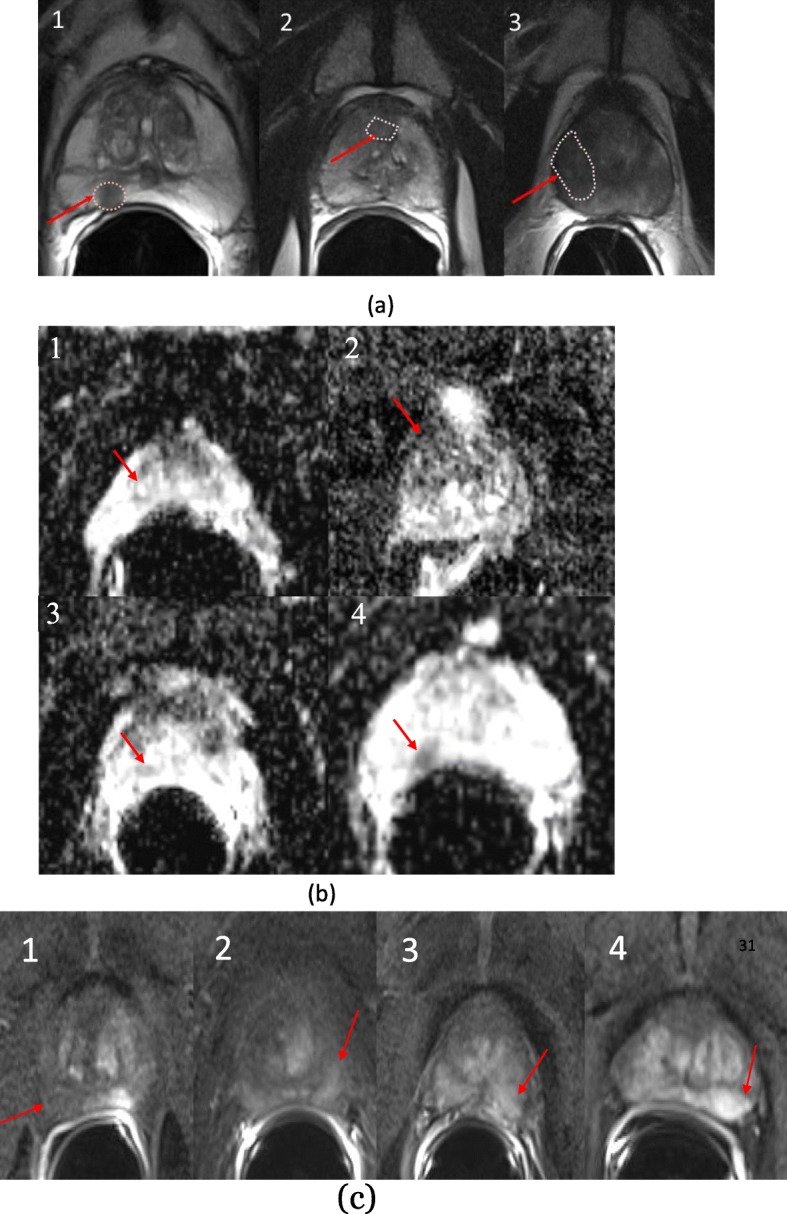
Example of semantic scoring for prostate cancer (**a**) Nodule shape / border, where (1 = round/ oval, 2 = irregular, 3 = amorphous), Border (1 = well defined, 2 = everything else between 1 and 3, 3 = poorly defined), (**b**) ADC intensity (1 = hyperintensity, 2 = iso-intensity, 3 = hypointensity, 4 = marked hypointensity), (**c**) Nodule enhancement (1 = no enhancement, 2 = slight enhancement, 3 = moderate enhancement, 4 = obvious enhancement)

Semantic descriptors were derived from lesions targeted for UroNav Fusion biopsy. The semantics were marked for each modality (T_2_WI, DCE, DWI, ADC) independently on a point scale (1 to 5). A total of 24 semantic features were developed, of that 16 were used in this study. Specifically, these features described the location, size, shape, margin, intensity and extra-prostatic extension of the lesion, the organ volume, and the presence of either benign prostate hyperplasia or lymphadenopathy (detailed explanation in Table 2). Figure 1 shows example patient MRI with semantic scores, where **1a** shows score for nodule/shape characteristics, oval nodule was scored as 1, irregular nodule was scored as 2, amorphous was scored as 3. Fig. 1b**,** shows example of semantic score on ADC images, where the nodule on left upper was hyper intense, right upper was iso-intense, left lower was hypo intense, and right lower was ‘marked hypo intense’. Figure 1c, shows contrast enhanced images, left (first panel) shows no early enhancement, received a score of 1, Followed by light enhancement (score = 2), moderate enhancement (score = 3), and obvious enhancement (score = 4) of the nodule indicated by an arrow, respectively.. The semantic features were systematically scored on a point scale (ranges from 2 levels up to 5) by the radiologists (Q.L. and H.L) and the PI-RADS version-2 (referred to as PI-RADS in this article) were independently evaluated following the guidelines of American College of Radiology (ACR). To assess the variability of the semantics among expert readers, we randomly selected 40 lesions (34 patients) and a third radiologist (J.C.) independently reviewed the scans and scored the semantics using the scoring sheet and point scale descriptors.

**Table 2:** Detailed semantic descriptors for prostate cancer a) broad categories b) feature description

a. Feature categories				
Prostate Semantics				
	Categories		Features	Description
1	Semantics		16	Shape, border, lymphadenopathy (F1:shape, F2:border, F3:T2 intensity, F4:ADC intensity, F5:homogeneity, F6:enhacement degree, F7:early enhancement, F8:enhanced homogenicity, F9:capsule, F10:cyst, F11:extraprostatic extension, F12:seminal vesicles, F13:distal sphincter, F14:bladder neck, F15:lymph adenopathy, F16:BPH)
2	PIRADS		1	Over-all
b. Semantic scoring sheet for prostate nodules (*Note:* level 0 to k was mapped to 1 to k + 1 with fixed offset).				
Characteristics			Definition	Scoring definition
lesion	Location	section	central location: nodule located in the central zone or transitional zone peripheral location: tumor located in the peripheral zone	1 = central gland 2 = peripheral zone 3 = both
		Lateral	The prostate is divided into right/left on axial sections by a vertical line drawn through the center	1 = L 2 = R 3 = Both
	Size	maximum transverse diameter	longest nodule width measured on axial images	
		maximum longitudinal diameter	Longest nodule length measured on sagittal images	
		maximum AP diameter	Longest nodule anterior-posterior diameter measuring on axial images	
	Shape		the overall shape of roundness	1 = round/ oval 2 = irregular 3 = amorphous
	Margin	Border definition	well or ill-defined border	1 = well defined 2 = everything else between 1 and 3 3 = poorly defined
		Capsule	Whether capsule could be found for the nodule	0 = absence 1 = presence
	intensity	rT2	The intensity of nodule on T2WI compared with the intensity of normal peripheral zone	T_2_WI _nodule_/T_2_WI _peri_
		rADC	The ADC value of nodule compared with that of normal peripheral zone	ADC _nodule_ /ADC _peri_
		T2 intensity	T2 signal intensity of the lesion was compared to surrounding tissues and defined as “marked hypointensity” if the lesion expressed similar signal intensity than back muscles, “hypointensity” if the lesion was brighter than back muscles but darker than adjacent prostate tissue, “iso” if the lesion was similar to adjacent prostate tissue, and “hyperintensity” if the lesion was brighter than the adjacent prostate tissue.	1 = hyperintensity 2 = iso-intensity 3 = hypointensity 4 = marked hypointensity
		Homogeneity on T2WI		0 = no 1 = yes
		Cyst	The presence or absence of cyst in the nodule	0 = absence 1 = presence
		ADC intensity		1 = hyperintensity 2 = iso-intensity 3 = hypointensity 4 = marked hypointensity
		Enhancement degree		1 = no enhancement 2 = slight enhancement 3 = moderate enhancement 4 = obvious enhancement
		Early enhancement		0 = absence 1 = presence
		Enhancement homogeneity	Nodule homogeneity after enhancement	0 = absence 1 = presence
	Extraprostatic extension	Extracapsular extension		0 = No signs of ECE 1 = Capsular abutment 3 = Capsular irregularity, retraction or thickening 4 = Neurovascular bundle thickening 4 = Bulge or loss of capsule 5 = Measurable extracapsular disease
		Seminal vesicle	Whether seminal vesicle is invaded by prostate tumor or not	0 = No signs of invasion 1 = Expansion 2 = Low T2 signal 3 = Filling in of angle 4 = Enhancement and impeded diffusion
		Bladder neck	Whether bladder neck is invaded by prostate tumor or not	2 = Adjacent tumor 3 = Loss of low T2 signal in bladder muscle 4 = Abnormal enhancement extending into bladder neck
		Distal sphincter	Whether distal sphincter is invaded by prostate tumor or not	2 = Adjacent tumor 3 = effacement of low signal sphincter muscle 4 = Abnormal enhancement extending into sphincter
prostate	volume		(maximum AP diameter) × (maximum transverse diameter) × (maximum longitudinal diameter) × 0.52	
	Benign prostate hyperplasia		The AP/transverse/ longitudinal diameter of prostate larger than 5 cm	0 = absence 1 = presence
other	Lymphadenopathy		The short axis of lymph node larger than 8 mm	0 = absence 1 = presence

### Statistical analysis

Agreement between the radiologists (Q.L. and J.C) was measured by the (weighted) Kappa index [25] for binary or ordinal variables. The kappa value was interpreted as follows: < 0, less than chance agreement; 0.01 to 0.2, slight agreement; 0.21 to 0.4, fair agreement; 0.41 to 0.6, moderate agreement; 0.61 to 0.8, substantial agreement; > 0.8, almost perfect agreement [26]. In our analysis, the radiologists scored 16 semantic features. Of these, 4 features had kappa value ≥0.7, 4 feature values were between kappa ≥0.6 and < 0.7, 4 features had kappa ≥0.5 and < 0.6, and 4 features could not be scored due to a limited range of the semantic characteristics (see Table 3).

**Table 3:** Reproducibility of Semantics features and PIRADS scored between two radiologists on randomly selected prostate patients with 40 targeted biopsies (32 unique patients). A) Actual scores b) sorted scores

Semantic Features			
#	Features	Kappa (CI)	
1	F1:shape	0.56 [0.33, 0.80]	
2	F2:border	0.74 [0.55, 0.93]	
3	F3:T2-intensity	0.58 [0.33, 0.84]	
4	F4:ADC-intensity	0.6 [0.369, 0.83]	
5	F5:homogeneity	0.55 [0.30, 0.81]	
6	F6:enhacement-degree	0.67 [0.48, 0.86]	
7	F7:early-enhancement	0.86 [0.66, 1.05]	
8	F8:enhanced-homogenicity	0.70 [0.47, 0.92]	
9	F9:capsule	0.54 [0.04, 1.04]	
10	F10:cyst	1 [1,1]	
11	F11:extraprostatic-extension	0.69 [0.49, 0.9]	
12	F12:seminal-vesicles	NA	
13	F13:distal-sphincter	NA	
14	F14:bladder-neck	NA	
15	F15:lymph-adenopathy	NA	
16	F16:BPH	0.72 [0.49, 0.95]	
PIRADS		0.69 [0.47, 0.90]	
	Kappa range	# Features	Details
1	≥0.7	4	F10:Cyst, F7:Early-enhancement, F2:Border,F16:BPH
2	≥0.6, < 0.7	4	F8:Enhanced-homogenic, F11:extraprostatic-extension, F6:enhacement-degree, F4:ADC-intensity
3	≥0.5, < 0.6	4	F3:T2-intensity, F1:Shape,F5:homogeneity, F9:capsule
4	NA (can’t evaluate)	4	F12:seminal-vesicles, F13:distal-sphincter;F14:bladder-neck, F15:lymph-adenopathy.

NA: Not enough examples to compute kappa score

We built a linear classifier model to find discriminant features that distinguish clinically significant cancers from indolent cases (GS ≥ 7 Vs GS ≤ 6), and indolent cases from benign (GS =6 Vs Benign). We selected the best 3 semantic features, taking all possible feature combinations ranked by Youden’s index [27, 28] for selecting highly predictive discriminators. The statistics were estimated following a cross validation approach (Hold out, 10 fold), randomly repeated over 100 times [29]. We also find the area under the receiver operator characteristics (AUROC) along with sensitivity, specificity, positive predictive value, and negative predictive value for the multivariable pairs of interest. The reported statistics were the ensemble value obtained over random repeats, and 95% confidence limits for the values reported.

## Results

**Fig. 2:**
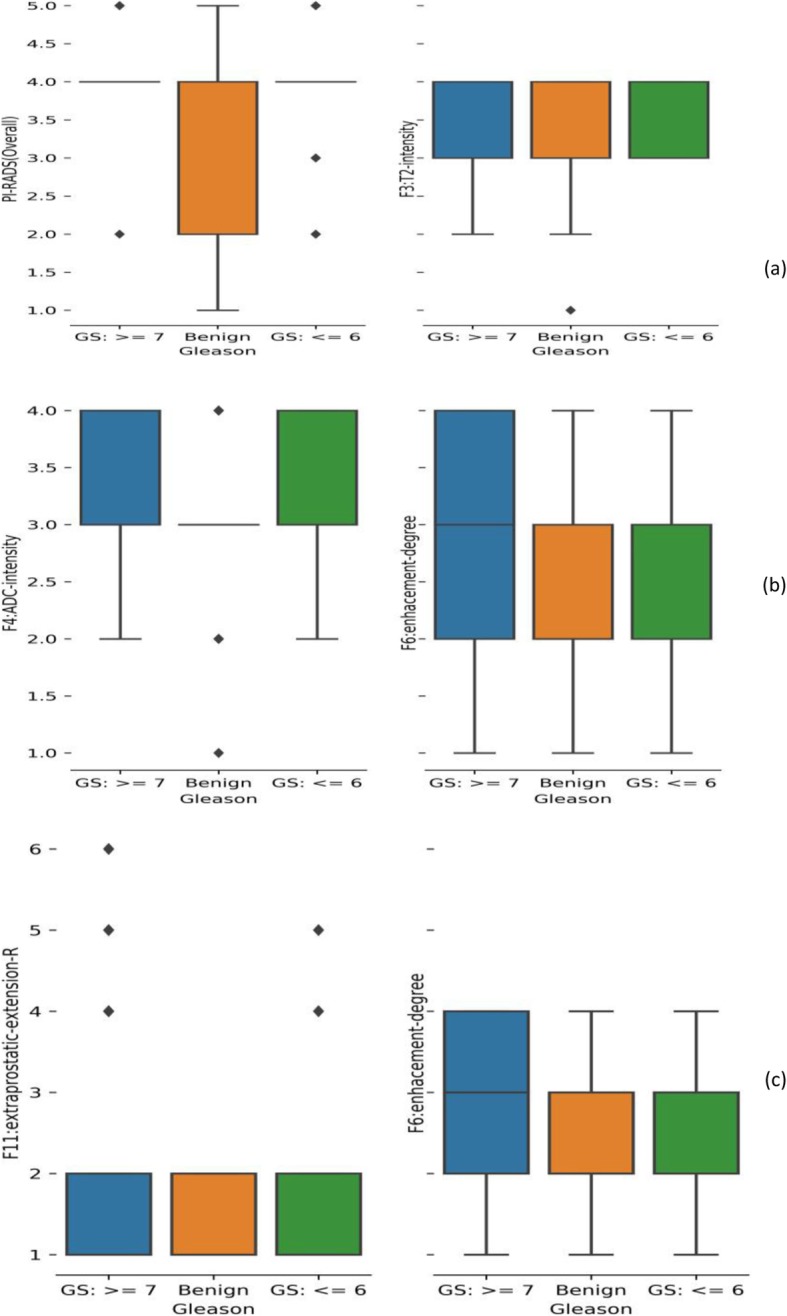
Box plot shows semantic traits across Gleason grades in the study cohorts. (**a**) PIRADS and T2 semantics trait, (**b**) ADC semantic trait and enhancement edge, (**c**) enhanced homogeneity and extra-prostatic extension

The final cohort used for the study had 167 biopsies (103 patients) with 57 biopsies that were considered clinically significant tumors (GS ≥ 7), 33 biopsies that were indolent tumors (GS ≤ 6), and 77 biopsies that were benign. Patient age ranged from 46 to 75 years at diagnosis. The PSA levels ranged from 0.8–44.7 ng/ml. Figure 2 shows distribution of semantic values in a box plot, with PI-RADS score plotted against pathological Gleason scores.

Among the semantic features, the Kappa scores for capsule status (presence or absence), homogeneity, shape, T2 intensity, ADC-intensity showed moderate agreement. Enhancement degree, extra-prostatic extension, enhanced homogeneity, and border were in substantial agreement between readers, while early enhancement and cyst (presence or absence) showed almost perfect agreement. The scores for four features, including seminal vesicle involvement, distal sphincter involvement, bladder neck involvement and lymphadenopathy, could not be computed, due to lack of examples.

We find ADC-intensity, homogeneity, and early enhancement to be univariate semantic predictors that gives the highest average AUROC (0.57 to 0.68). The PPV (positive predictive value) and sensitivity for these markers are relatively high, with average values to be [0.62 to 0.69] and [0.82 to 0.96] respectively, for finding the clinically significant prostate cancers (GS ≥ 7).

When these features were combined together, we found the combination of ADC-intensity, T2-Intensity, and enhancement homogeneity showed the highest average AUROC of 0.70, with average sensitivity and PPV for detecting the aggressive cancer to be 0.79 and 0.72, respectively. The next best feature combination was based on early ADC intensity, Border, enhancement homogeneity, that had an average AUROC of 0.71, with average sensitivity and PPV for detecting aggressive cancer to be 0.82 and 0.68, respectively. In comparison, we characterized predictors that discriminate aggressive from indolent prostate cancers using overall PI-RADS (version 2) scores. We repeated the predictive analysis with different level of cutoffs on the PI-RADS scores to discriminate aggressive cancer (i.e. PI-RADS≥5, PI-RADS≥4, PI-RADS≥3). We found that having a moderate cutoff (PI-RADS≥4) showed the highest AUROC of 0.6, with sensitivity and PPV of 0.98 and 0.69 respectively. Table 4 shows discriminant semantic features with their predictive statistics. We also find the top semantic predictors (ADC-intensity, T2-intensity, enhancement homogeneity) receiver operator characteristics was significantly different from PI-RADS3 (*p* = 0.0022), PI-RADS5 (*p* = 0.0048) based predictor of malignancy defined by Gleason score (GS ≥7). While semantics predictor was non-significant with PI-RADS4 (*p* = 0.0724) predictor, where significance was computed using non-parametric Delong’s statistics [30].

**Table 4:** Features based predictors that discriminate aggressive grade (Gleason ≥7 Vs ≤ 6) prostate cancers a) univariate semantic predictors b) multivariable semantic predictors (up to 3 semantics) c) PIRADS based predictor

	Features	Error	Sensitivity/Specificity	PPV/NPV	E [AUC],σ, [CI]
a) Single Predictive Semantic: ≥ GS 7 Vs =6 GS || Samples: 90 (57 Vs 33)					
1	F4:ADC-intensity	0.384	0.821/0.342	0.687/0.505	0.678 (0.114) [0.401,0.905]
2	F8:enhanced-homogenicity-R	0.379	1/0	0.636/0	0.583 (0.099) [0.356,0.765]
3	F7:early-enhancement-R	0.393	0.96/0.027	0.618/0.014	0.57 (0.116) [0.281,0.758]
4	F11:extraprostatic-extension-R	0.378	1/0	0.628/0	0.542 (0.115) [0.314,0.749]
5	F3:T2-intensity	0.396	0.965/0.016	0.637/0.005	0.549 (0.119) [0.254,0.751]
b) Multivariable Semantic Predictor: ≥ GS 7 Vs =6 GS || Samples: 90 (57 Vs 33)					
1	F3:T2-intensity;F4:ADC-intensity: F8:enhanced-homogenicity-R	0.333	0.793/0.465	0.719/0.576	0.701 (0.124), [0.426,0.94]
2	F4:ADC-intensity;F8:enhanced-homogenicity-R:F11:extraprostatic-extension-R	0.329	0.799/0.464	0.73/0.557	0.687 (0.118), [0.46,0.94]
3	F2:border;F4:ADC-intensity: F8:enhanced-homogenicity-R	0.334	0.819/0.412	0.681/0.6	0.706 (0.095), [0.474,0.886]
4	F4:ADC-intensity;F5:homogeneity-R:F8:enhanced-homogenicity-R	0.341	0.808/0.418	0.712/0.553	0.698 (0.118), [0.457,0.948]
c) PIRADS as a Predictor: ≥ GS 7 Vs = 6 GS || Samples: 90 (57 Vs 33)					
1	PIRADS (> = 3 Cancer)	0.34 (0.096)	0.981/0.104	0.654/0.483	0.542 (0.06), [0.458,0.701]
2	PIRADS (> = 4 Cancer)	0.296 (0.091)	0.981/0.22	0.686/0.717	0.6 (0.08), [0.462,0.752]
3	PIRADS (> = 5 Cancer)	0.531 (0.101)	0.22/0.893	0.752/0.4	0.557 (0.083), [0.371,0.707]

We then built models to find semantic predictors to differentiate indolent (GS =6) from benign cases. We found extra-prostatic extension, early enhancement, ADC intensity features to be univariate discriminators, with an average AUROC of 0.58 to 0. 61. When combining these features together, the combination of ADC intensity, early enhancement and extra prostatic extension shows the highest average AUROC of 0.63 with an average sensitivity and PPV of 0.16 and 0.51 respectively. The next feature combination of homogeneity, early enhancement degree, and extra prostatic extension had an average AUROC of 0.63, with sensitivity and PPV of 0.20 and 0.57 respectively.

**Fig. 3:**
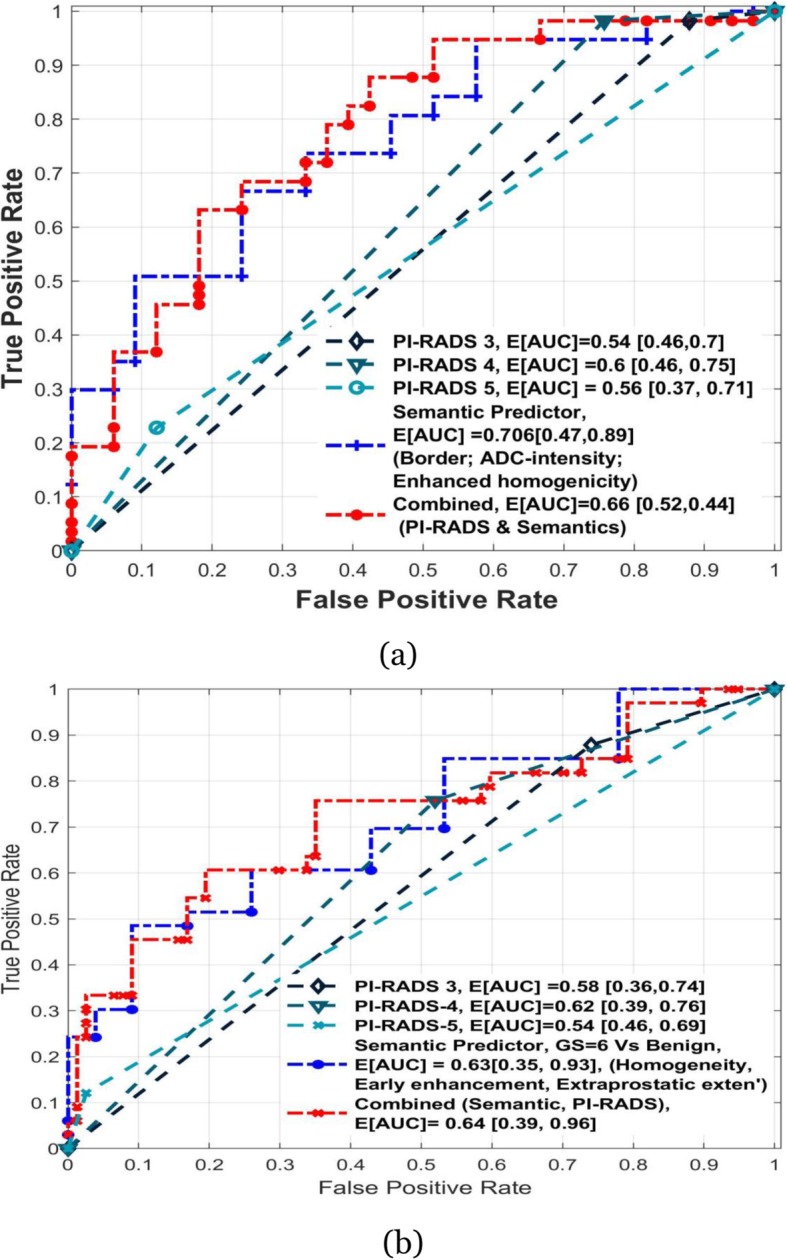
Receiver operator characteristic of semantic & PI-RAD based predictors (**a**) identify clinically significant grade prostate cancer (≥ GS7 from GS 6) and (**b**) Gleason 6 from benign

Receiver operator characteristics for top predictors is show in Fig. 3**.** Adding semantics to PI-RADS increases average AUC to 0.64 from 0.63 for GS6 vs Benign and lowers from 0.7 to 0.66 for GS 7 Vs GS6.

## Discussion

In this study, we propose a radiological semantic scheme that captures traits on a point scale independently on different modalities of mpMRI. We used the semantic descriptor as a combination to build linear discriminant functions to identify clinically significant prostate cancers. These semantic predictors were then compared to PI-RADS-v2 based discriminators, the American College of Radiology had adopted the use of PI-RADS (version 2) system to report standardized prostate cancer findings in mpMRI [17, 31]. We found that semantics demonstrated better predictability of pathological outcome compared to PI-RADS based predictors. We believe semantic traits may help reduce the variability in image interpretation between radiologists as the observational scorings are made for a trait, independently in a modality (T2w, ADC, DCE). Semantics scoring is specifically defined to obtain an expert opinion about a radiological trait, such as the presence or absence of a trait, or the multi-level appearance of a trait in the scan.

In a recent report PI-RADS 1 and 2 scoring schemes were compared and report a PPV of 75% for both versions to find clinically significant cancers. The NPV (negative predictive value) was 46% for PI-RADS-1 and 43% for PI-RADS-2, in a cohort of 66 patients [32].

Tissue cell densities have been well characterized and is reflective of molecular movement, in prostate carcinoma it is, characterized by reduced ADC values [33]. Further, ADC value in prostate has been shown to be related to Gleason score showing an inverse trend [34, 35]. It is useful in differentiating carcinoma from benign hyperplasia [36], high-risk patients from those at low and intermediate risk [37] and helpful for transitional zone (TZ) lesion detection [38]. We also find that ADC is a critical marker in identifying clinical significant cancer and are capabale of distinguishing indolent from benign cases. Due to interpretational variability of dynamic contrast enhancement images, they do not contribute to the overall clinical assessment of prostate lesions, especially in PIRADS-v2 (exception of PI-RADS score of 3). While in our study, early enhancement and enhancement degree were effective predictors, and the cancerous nodule usually presents early enhancement and higher enhancement degree. When combined with ADC intensity and extra-prostatic extension, they form better predictors of clinically significant cancers.

. Clinically, any non-binary point scale can lead to some level of unnecessary confusion to practitioners, and eventually leading to variability in diagnosis that will impact the patient care [39]. In our study, we used discriminator functions and formed different multivariable models agnostically combing traits across modalities, with each trait having equal likelihood to be part of the predictor model. We limit the size of the predictors to three semantic traits due to a limited sample size. This approach allows combination of information across modalities to find clinically significant prostate cancers.

We find PI-RADS based predictor with a cutoff of ≥4 showed slightly lower discriminatory ability to find clinically significant cancers (AUROC of 0.6, sensitivity and PPV of 0.98 and 0.68 respectively), compare to its ability to differentiate indolent from benign (AUROC of 0.62, PPV of 0.38 and Sensitivity of 0.77). We find semantics based predictors shows better performance, with an AUROC of 0.70 and 0.63 for discriminating clinically significant versus indolent tumor and indolent tumor versus benign, respectively (see Table 4 & 5). We also find adding semantics to PI-RADS (overall score) shows improvement in predictor performance, both in discriminating clinically significant lesion (GS ≥ 7) from indolent (GS =6) and benign from indolent (GS =6).

**Table 5:** Features based predictors that discriminate indolent grade cancer (Gleason = 6 Vs Benign) from benign a) univariate semantic predictors b) multivariable semantic predictors (up to 3 semantics) c) PIRADS based predictor

	Features	Error	Sensitivity/Specificity	PPV/NPV	E [AUC],σ, [CI]
A) Single Predictive Semantic: Gleason 6 Vs Benign || Samples: 110 (33 Vs 77)					
1	F11:extraprostatic-extension-R	0.279	0.058/1	0.34/0.71	0.586 (0.12) [0.307,0.822]
2	F7:early-enhancement-R	0.301	0/1	0/0.688	0.609 (0.1) [0.391,0.842]
3	F4:ADC-intensity	0.319	0.014/0.98	0.006/0.702	0.614 (0.119) [0.348,0.928]
4	F6:enhacement-degree	0.31	0.026/0.982	0.049/0.706	0.562 (0.126) [0.328,0.815]
5	F3:T2-intensity	0.308	0/1	0/0.701	0.546 (0.103) [0.35,0.775]
B) Multivariable Semantic Predictor: GS 6 Vs Benign || Samples 110 (33 Vs 77)					
1	F5:homogeneity-R;F7:early-enhancement-R:F11:extraprostatic-extension-R	0.287	0.204/0.954	0.571/0.735	0.627 (0.137), [0.347,0.931]
2	F4:ADC-intensity;F7:early-enhancement-R:F11:extraprostatic-extension-R	0.295	0.156/0.954	0.514/0.725	0.632 (0.114), [0.385,0.822]
3	F3:T2-intensity;F7:early-enhancement-R:F11:extraprostatic-extension-R	0.305	0.157/0.947	0.404/0.743	0.624 (0.136), [0.406,0.909]
4	F5:homogeneity-R;F7:early-enhancement-R:F11:extraprostatic-extension-R	0.287	0.204/0.954	0.571/0.735	0.627 (0.137), [0.347,0.931]
C) PIRADS as a Predictor (GS 6 Vs Benign) || Samples: 110 (33 Vs 77)					
1	PIRADS(≥ 3)	0.544	0.878/0.271	0.344/0.839	0.575 (0.083) [0.358,0.744]
2	PIRADS(≥ 4)	0.44	0.769/0.468	0.384/0.823	0.618 (0.094) [0.393,0.775]
3	PIRADS(≥ 5)	0.286	0.102/0.978	0.449/0.716	0.54 (0.062) [0.46,0.689]

There is a high level of subjectivity among radiologists in scoring PI-RADS (v.2) [40], in a recent review, these shortcomings were categorized into clinical indications and technical/physiological artifacts [41, 42]. The clinical consequence in disease identification has resulted in impacting patient care by over-detection in some cases and missed diagnosis of aggressive cancer in others. We believe evaluation of semantic traits in mpMRI images will reduce subjectivity in tumor detection.

In our study, trained radiologists were asked to describe observed traits on a point scale following the semantic descriptors and these are then related to pathological outcome. The use of semantic discriminant functions may provide an alternative real value risk score to the oncologist to decide upon an appropriate management plan for the patient. We understand that there is a further need to train such predictors on a larger cohort to obtain balanced coefficients based on the radiological traits.

We believe semantic predictors can discriminate clinically significant cancers and provide valuable risk assessment to aid clinical decisions both in targeting lesions and planning treatment for the disease.

### Limitations

- *We have assembled over 103 patients (167 biopsies) all of the data was obtained in a single institution with diverse cohort and used to train the model in cross validation setting. The data in our center were obtained from couple of clinical locations and biopsies carried out by multiple urologists. Data from multi-institutions will improve diversity of the cohort.*
- *This approach will have a better possibility of obtaining a stable model with independent test and validation cohort. We acknowledges the absence of such a dataset.*
- *We used the lesions on mpMRI scan to make semantic assessment and pathological validation was obtained by TRUS/MPI biopsy. It’s possible that core lesion may have been missed leading tumor, leading to lower gleason grade, consequentially reduce classifier performance.*

## Conclusions

The proposed radiological semantic schema to describe prostate lesions on mpMPI shows promise in quantifying tumor imaging traits. A model based approach of these traits provides a computational means to relate these findings to pathological outcome. These methods show potential in discriminating prostate cancer lesions with better accuracy than currently practiced risk assessment.

## Data Availability

The patient images (mpMRI) and pathological diagnosis are part of standard of care and reside at the institution. Part of the data will be de-identified and available through public portal on request.

## Abbreviations

3D: 3-dimensions
ADC: Apparent Diffusion Coefficient of MRI
BK-US: BK Ultrasound Fusion System
CAD: Computer Aided Diagnosis
DCE: Dynamic Contrast Enhancement Images
GS: Gleason Score
H&E: Hemotoxylin and Eosin
mpMRI or MRI: Multi-parametric Magnetic Resonant Imaging
PACS: Picture archive and communication system
PSA: Prostate Specific Antigen
T2w: T2-weighted MRI
TRUS Biopsy: Transrectal Ultrasound Biopsy system

## References

[1] Siegel RL, Miller KD, Jemal A (2018). Cancer statistics, 2018. CA Cancer J Clin.

[2] Schröder FH, Hugosson J, Roobol MJ (2009). Screening and prostate-cancer mortality in a randomized European study. New Engl J Med.

[3] Draisma G, Etzioni R, Tsodikov A (2009). Lead time and Overdiagnosis in prostate-specific antigen screening: importance of methods and context. Yearbook Urol.

[4] Porten SP, Whitson JM, Cowan JE (2011). Changes in prostate cancer grade on serial biopsy in men undergoing active surveillance. J Clin Oncol.

[5] Westhoff N, Siegel FP, Hausmann D (2017). Precision of MRI/ultrasound-fusion biopsy in prostate cancer diagnosis: an ex vivo comparison of alternative biopsy techniques on prostate phantoms. World J Urol.

[6] Siddiqui MM, Rais-Bahrami S, Turkbey B (2015). Comparison of MR/ultrasound fusion-guided biopsy with ultrasound-guided biopsy for the diagnosis of prostate cancer. Jama.

[7] Dickinson L, Ahmed HU, Allen C (2011). Magnetic resonance imaging for the detection, localisation, and characterisation of prostate cancer: recommendations from a European consensus meeting. Eur Urol.

[8] Heidenreich A (2011). Consensus criteria for the use of magnetic resonance imaging in the diagnosis and staging of prostate cancer: not ready for routine use. Eur Urol.

[9] Seo JW, Shin S-J, Taik Oh Y (2017). PI-RADS version 2: detection of clinically significant cancer in patients with biopsy Gleason score 6 prostate cancer. Am J Roentgenol.

[10] Valerio M, Donaldson I, Emberton M (2015). Detection of clinically significant prostate cancer using magnetic resonance imaging-ultrasound fusion targeted biopsy: a systematic review. Eur Urol.

[11] Schiebler ML, Yankaskas BC, Tempany C (1992). MR imaging in adenocarcinoma of the prostate: interobserver variation and efficacy for determining stage C disease. AJR Am J Roentgenol.

[12] Villers A, Puech P, Mouton D, Leroy X, Ballereau C, Lemaitre L (2006). Dynamic contrast enhanced, pelvic phased array magnetic resonance imaging of localized prostate cancer for predicting tumor volume: correlation with radical prostatectomy findings. J Urol.

[13] Jung JA, Coakley FV, Vigneron DB (2004). Prostate depiction at endorectal MR spectroscopic imaging: investigation of a standardized evaluation system. Radiology.

[14] Arumainayagam N, Kumaar S, Ahmed HU (2010). Accuracy of multiparametric magnetic resonance imaging in detecting recurrent prostate cancer after radiotherapy. BJU Int.

[15] Dickinson L, Ahmed HU, Allen C (2013). Scoring systems used for the interpretation and reporting of multiparametric MRI for prostate cancer detection, localization, and characterization: could standardization lead to improved utilization of imaging within the diagnostic pathway?. J Magn Reson Imaging.

[16] Barentsz JO, Weinreb JC, Verma S (2016). Synopsis of the PI-RADS v2 guidelines for multiparametric prostate magnetic resonance imaging and recommendations for use. Eur Urol.

[17] American-College-of-Radiology (2015). MR Prostate Imaging Reporting and Data System version 2.

[18] Rosenkrantz AB, Ginocchio LA, Cornfeld D (2016). Interobserver reproducibility of the PI-RADS version 2 lexicon: a multicenter study of six experienced prostate radiologists. Radiology.

[19] Schimmöller L, Quentin M, Arsov C (2013). Inter-reader agreement of the ESUR score for prostate MRI using in-bore MRI-guided biopsies as the reference standard. Eur Radiol.

[20] Steiger P, Thoeny HC (2016). Prostate MRI based on PI-RADS version 2: how we review and report. Cancer Imaging.

[21] Kumar V, Gu Y, Basu S (2012). Radiomics: the process and the challenges. Magn Reson Imaging.

[22] Balagurunathan Y, Gu Y, Wang H (2014). Reproducibility and prognosis of quantitative features extracted from CT images. Transl Oncol.

[23] Gillies RJ, Kinahan PE, Hricak H (2016). Radiomics: images are more than pictures, they are data. Radiology.

[24] Parmar C, Grossmann P, Bussink J, Lambin P, Aerts HJWL (2015). Machine learning methods for quantitative radiomic biomarkers. Sci Rep.

[25] Sim J, Wright CC (2005). The kappa statistic in reliability studies: use, interpretation, and sample size requirements. Phys Ther.

[26] Viera AJ, Garrett JM (2005). Understanding interobserver agreement: the kappa statistic. Fam Med.

[27] Youden W (1950). Index for rating diagnostic tests. Cancer.

[28] Ruopp MD, Perkins NJ, Whitcomb BW, Schisterman EF (2008). Youden index and optimal cut-point estimated from observations affected by a lower limit of detection. Biom J.

[29] Hua Jianping, Balagurunathan Yoganand, Chen Yidong, Lowey James, Bittner Michael L., Xiong Zixiang, Suh Edward, Dougherty Edward R. (2006). Normalization Benefits Microarray-Based Classification. EURASIP Journal on Bioinformatics and Systems Biology.

[30] DeLong ER, DeLong DM, Clarke-Pearson DL (1988). Comparing the areas under two or more correlated receiver operating characteristic curves: a nonparametric approach. Biometrics.

[31] Vargas HA, Hotker AM, Goldman DA (2016). Updated prostate imaging reporting and data system (PIRADS v2) recommendations for the detection of clinically significant prostate cancer using multiparametric MRI: critical evaluation using whole-mount pathology as standard of reference. Eur Radiol.

[32] Aliukonis P, Letauta T, Briediene R, Naruseviciute I, Letautiene S (2017). The role of different PI-RADS versions in prostate multiparametric magnetic resonance tomography assessment. Acta Med Lituanica.

[33] Zelhof B, Pickles M, Liney G (2009). Correlation of diffusion-weighted magnetic resonance data with cellularity in prostate cancer. BJU Int.

[34] Łuczyńska E, Heinze-Paluchowska S, Domalik A (2014). The utility of diffusion weighted imaging (DWI) using apparent diffusion coefficient (ADC) values in discriminating between prostate cancer and normal tissue. Med Sci Monit Basic Res.

[35] Kim TH, Jeong JY, Lee SW (2015). Diffusion-weighted magnetic resonance imaging for prediction of insignificant prostate cancer in potential candidates for active surveillance. Eur Radiol.

[36] Oto A, Kayhan A, Jiang Y (2010). Prostate cancer: differentiation of central gland cancer from benign prostatic hyperplasia by using diffusion-weighted and dynamic contrast-enhanced MR imaging. Radiology.

[37] Caivano R, Rabasco P, Lotumolo A (2013). Comparison between Gleason score and apparent diffusion coefficient obtained from diffusion-weighted imaging of prostate cancer patients. Cancer Investig.

[38] Yoshimitsu K, Kiyoshima K, Irie H (2008). Usefulness of apparent diffusion coefficient map in diagnosing prostate carcinoma: correlation with stepwise histopathology. J Magn Reson Imaging.

[39] Richenberg JL (2016). PI-RADS: past, present and future. Clin Radiol.

[40] Kitzing YX, Prando A, Varol C, Karczmar GS, Maclean F, Oto A (2016). Benign conditions that mimic prostate carcinoma: MR imaging features with histopathologic correlation. Radiographics.

[41] Panebianco V, Giganti F, Kitzing YX (2018). An update of pitfalls in prostate mpMRI: a practical approach through the lens of PI-RADS v. 2 guidelines. Insights Into Imaging.

[42] Rosenkrantz AB, Taneja SS (2014). Radiologist, be aware: ten pitfalls that confound the interpretation of multiparametric prostate MRI. AJR Am J Roentgenol.

